# Effect of High Protein Diet and Probiotic* Lactobacillus casei* Shirota Supplementation in Aflatoxin B_1_-Induced Rats

**DOI:** 10.1155/2018/9568351

**Published:** 2018-05-23

**Authors:** Z. Nurul Adilah, Winnie-Pui-Pui Liew, S. Mohd Redzwan, I. Amin

**Affiliations:** Department of Nutrition and Dietetics, Faculty of Medicine and Health Sciences, Universiti Putra Malaysia, 43400 Serdang, Selangor, Malaysia

## Abstract

Probiotic* Lactobacillus casei* Shirota (LcS) is a potential decontaminating agent of aflatoxin B_1_ (AFB_1_). However, few studies have investigated the influence of diet, especially a high protein (HP) diet, on the binding of AFB_1_ by probiotics. This research was conducted to determine the effect of HP diet on the ability of LcS to bind AFB_1_ and reduce aflatoxin M_1_ (AFM_1_) in AFB_1_-induced rats. Sprague Dawley rats were randomly divided into three groups: A (HP only), B (HP + 10^8^ CFU LcS + 25 *μ*g AFB1/kg BW), and C (HP + 25 *μ*g AFB1/kg BW). Levels of AST and ALP were higher in all groups but other liver function's biomarkers were in the normal range, and the liver's histology showed no structural changes. The urea level of rats in group B (10.02 ± 0.73 mmol/l) was significantly lower (*p* < 0.05) than that of rats in group A (10.82 ± 0.26 mmol/l). The presence of carcinoma in the small intestine and colon was more obvious in group C than in group B. Moreover, rats in group B had significantly (*p* < 0.05) lower AFM_1_ concentration (0.39 ± 0.01 ng/ml) than rats in group C (5.22 ± 0.28 ng/ml). Through these findings, LcS supplementation with HP diet alleviated the adverse effects of AFB_1_ by preventing AFB_1_ absorption in the small intestine and reducing urinary AFM_1_.

## 1. Introduction

Aflatoxin is produced by* Aspergillus flavus* and* Aspergillus parasiticus* [1]. This food contaminant can be found commonly in agricultural and food commodities such as maize, grains, peanuts, cereal, and animal feeds [1]. Food legislation and food processing are becoming more advanced, yet they are still unable to prevent the occurrence of aflatoxin in food commodities. Due to high stability of aflatoxin, it has become a problem not only during harvesting but also at every stage of food production, starting from harvesting of raw material, storage, and processing until the food reaches the consumers [2]. Aflatoxin should be removed, as long-term consumption of aflatoxin-contaminated food can cause carcinogenic and toxicity effects on humans and animals [3].

One of the main adverse effects of dietary aflatoxin exposure is aflatoxicosis. Aflatoxicosis is a foodborne disease due to aflatoxin ingestion in the diet and it can be categorized into acute and chronic aflatoxicosis. Acute aflatoxicosis results in death, whereas chronic aflatoxicosis can cause immune suppression, cancer, and other “slow” pathohistological conditions [4]. The liver is the main target of aflatoxicosis. In fact, one of the metabolites of aflatoxin, aflatoxin B_1_ (AFB_1_), has been classified by the International Agency for Research on Cancer (IARC) as group 1 carcinogen and is linked to the development of the liver cancer [5].

The recent alternative approach to remove AFB_1_ from the body is through the consumption of probiotic bacteria, as some studies have shown that probiotic bacteria might be a potential adsorbent of aflatoxin in the gastrointestinal tract by reducing aflatoxin bioavailability [6, 7]. World Health Organization (WHO) defined probiotics as “live microorganisms” which are able to provide advantages to the host when consumed in an adequate amount [8]. It is evident that probiotic consumption improved gastrointestinal health and immune system [9]. As an adsorbent of aflatoxin, probiotic bacteria remove AFB_1_ through noncovalent binding of AFB_1_ molecule to the bacterial cell wall [10]. Besides, polysaccharides and peptidoglycan components as well as teichoic acids of bacterial cell wall are involved in the binding process of AFB_1_ by probiotics [7]. The authors indicated also that teichoic acids play a major role in the binding of AFB_1_ by* Lactobacillus reuteri* and* Lactobacillus casei* Shirota (LcS) [7].

A randomized, double-blind, cross-over, placebo-controlled study with two 4-week intervention phases was conducted [11] to investigate the effectiveness of probiotics in reducing circular production of aflatoxin biomarkers in a population exposed to aflatoxin. The authors found that probiotic intervention reduced AFB_1_-lysine adduct (AFB_1_-lys) and urinary aflatoxin M_1_ (AFM_1_) in certain subjects [11]. Interestingly, it was found that diets may be one of the confounding factors that can affect the ability of probiotic LcS to bind AFB_1_ in the gastrointestinal tract. In addition, high intake of macronutrients can influence the metabolism of aflatoxin and subsequently affect the circular production of AFB_1_ metabolites [11].

In a recent review article [12], the authors mentioned that a high protein (HP) diet can affect aflatoxicosis. For example, rats fed with HP diet and exposed to AFB_1_ had no focal hyperplasia and less ductular reaction of liver [13]. These symptoms are commonly observed in the early stages of hepatocellular carcinoma (HCC). As such, the findings [13] may explain the effect of diets on the metabolism of aflatoxin as reported by Mohd Redzwan et al. [11] and to some extent slow the progression of HCC associated with aflatoxin exposure. On the other hand, it is still unclear whether diet manipulation can have influence on the activity of probiotics in reducing AFB_1_ bioavailability. A study by Nikbakht Nasrabadi et al. [14] found that LcS supplementation in aflatoxin-induced rats reduced serum AFB_1_. Nevertheless, the authors [14] did not take into consideration the influence of diet in the study protocol. Furthermore, diet was found as a confounding factor that can affect LcS activity and aflatoxin metabolism [11]. Therefore, this research was conducted to elucidate the effect of HP diet on the ability of probiotic LcS to reduce urinary AFM_1_ and certain aflatoxicosis symptoms in AFB_1_-induced rats.

## 2. Materials and Methods

Yakult fermented milk drink was purchased from a local supermarket in Serdang, Selangor, Malaysia. AFB_1_ was acquired from Trilogy Analytical Laboratory, Inc. (Vossbrink Drive, Washington). MRS broth, MRS agar, and sodium chloride were purchased from Merck (Darmstadt, Germany). Glycerol solution was procured from Sigma-Aldrich (St. Louis, MO, USA). ELISA kit for the detection of AFM_1_ in urine was purchased from Helica Biosystems, Inc. (Santa Ana, CA, USA).

### 2.1. Culturing Probiotic Bacteria

The source of LcS was from the Yakult cultured milk drink (contained live LcS) [14]. One hundred microliters (100 *μ*L) of Yakult was aseptically spread onto MRS agar and incubated for 48 hours aerobically at 37°C. Then, one colony of LcS was transferred into MRS broth and further cultured for another 24 hours aerobically at 37°C. The growth of LcS was recorded every two hours, and the corresponding CFU was monitored optically at 600 nm. One hundred microliters (100 *μ*L) was withdrawn from the broth and spread on the MRS agar to acquire the CFU. LcS's ability to bind AFB_1_ also depends on bacterial concentration. For a significant removal of 50% of aflatoxin, the minimum concentration of* Lactobacillus* bacteria needed is 2 × 10^9^ CFU/ml [14]. Based on the absorbance value (OD) and CFU that were recorded during the 24 h of incubation, the bacterial cell was incubated at least 19 h in order to reach concentration of 10^9^ CFU. The bacterial cells were harvested via centrifugation at 2200*g* using Kubota 2810 centrifuge (Tokyo, Japan) for 15 minutes and the supernatant was discarded. The bacterial pellet was resuspended with 50% (v/v) glycerol [14]. Prior to the usage, the glycerol liquid was replaced with saline solution. The identity of LcS cultured from Yakult cultured milk drink was confirmed with the 16S rRNA sequencing service provided by First BASE Laboratories Sdn Bhd (Seri Kembangan, Selangor, Malaysia).

### 2.2. Animals

Twenty-four (*n* = 24) male Sprague Dawley rats (7-8 weeks old, 290–300 g) were purchased from the Animal Resource Unit (ARU), Department of Veterinary Pathology and Microbiology, Faculty of Veterinary Medicine, University Putra Malaysia (UPM). The rats were kept at room temperature under standard conditions of light (12 h light-dark cycle) and regulated temperature (20–22°C) and ventilation in the animal research house of Comparative Medicine and Technology Unit (COMeT), Institute of Bioscience, UPM. Two rats were housed in a cage with wood shavings. The cleaning process of the cages was performed two times per week and water supply was changed on a daily basis. Ethical approval for this animal study was given by the Institutional Animal Care and Use Committee, UPM (UPM/IACUC/AUP-R098/2016).

### 2.3. Preparation of Diet

A high protein (HP) diet was prepared based on Envigo recipe [15]. This diet was approximately 40% protein in terms of calories.

### 2.4. Experimental Study

Twenty-four rats (*n* = 24) were randomly divided into three groups of diets. Rats in group A (*n* = 8) received HP diet only (HP only); rats in group B (*n* = 8) were fed with HP diet supplemented with LcS (10^8^-10^9^ CFU) and AFB_1_ (LcS + HP + AFB_1_), while rats in group C (*n* = 8) were only provided with HP diet and AFB_1_ (HP + AFB_1_). For rats in group B, immediately after the fifth probiotic dose, AFB_1_ was given at a complete dosage. The complete dosage of AFB_1_ for rats in group B was 25 *μ*g AFB_1_/kg body weight (BW). The dose selected in this study is equivalent to 0.03 to 0.45 mg/kg (30–450 ppb) of AFB_1_ in food. This range is commonly found in contaminated foods that are consumed daily by many populations, especially in developing countries [16, 17]. The rats were dosed five days per week and sacrificed 24 h following the last dose [16]. As for the rats in group C (*n* = 8), they were fed with HP diet and given the same AFB_1_ dose as previously described. Since rats of group A and C were not supplemented with LcS, they were gavaged with a saline solution. The repeated dose of AFB_1_ given in this study is a standard protocol for an animal study [18]. Overall, the experiment ran for 25 days and HP diet and water were provided ad libitum. Water and diet consumption as well as the body weight of all rats were recorded at the beginning and every three days. The body weight of rats was recorded using an electronic balance (A&D Co., Ltd., Tokyo, Japan). At the end of the intervention, all rats were anesthetized using Ketamine and Xylazine and blood sample was taken by cardiac puncture from the artery.

### 2.5. Urine Collection

Following the last dose of AFB_1_, all rats were kept individually in metabolic cage for the collection of urine. The urine samples were then stored at −80°C until analysis.

### 2.6. Blood Withdrawal

About 3–10 ml of blood was withdrawn and collected using blood collection tube with serum separator (Becton, Dickinson and Company (BD), Plymouth, UK). The blood serum was separated using Kubota 2810 centrifuge (Tokyo, Japan) at 4°C for 13 min at 2000*g*. Serum was collected and stored at −80°C until analysis.

### 2.7. Liver and Kidney Function Test

The levels of alanine aminotransferase (ALT), aspartate aminotransferase (AST), alkaline phosphatase (ALP), total protein, and albumin were measured for liver function, while the levels of urea (UREA) and blood creatinine (CREA) were measured to assess kidney function. These tests were analyzed using fully automated clinical analyzer BiOLiS 24i Premium at the Haematology and Biochemistry Clinical Laboratory of Faculty of Veterinary Medicine, UPM.

### 2.8. Analysis of Urinary AFM_1_

Urine was analyzed for the presence of AFM_1_ using ELISA kit, specifically for the determination of urinary AFM_1_ (Helica Biosystems, Inc., Santa Ana, CA, USA) [19].

### 2.9. Histopathological Examination

The entire small intestine, colon, liver, and spleen were removed and fixed in formalin solution 10%, Neutral Buffered (R&M Chemicals, UK), for 3 days at room temperature. Fixed tissue samples were washed several times with 80–95% ethanol, followed by dehydration in absolute ethanol before clearing with xylene and embedding in paraffin. The paraffin-embedded tissues were sectioned serially at 4 *μ*m thickness. The sections were stained with hematoxylin and eosin (H&E) for qualitative histological analysis.

### 2.10. Statistical Analysis

Data were analyzed using SPSS version 20 software (SPSS Inc., Chicago, IL). The mean differences of liver and kidney biomarkers were analyzed using ANOVA between groups and post hoc analysis (Tukey's test) was conducted for every significant ANOVA output. On the other hand, the difference of urinary AFM_1_ level was determined by independent *t*-test. Results were expressed in terms of mean ± SD. The level of significance was assigned at *p* < 0.05.

## 3. Result and Discussion

### 3.1. Body Weight Gain

**Figure 1:**
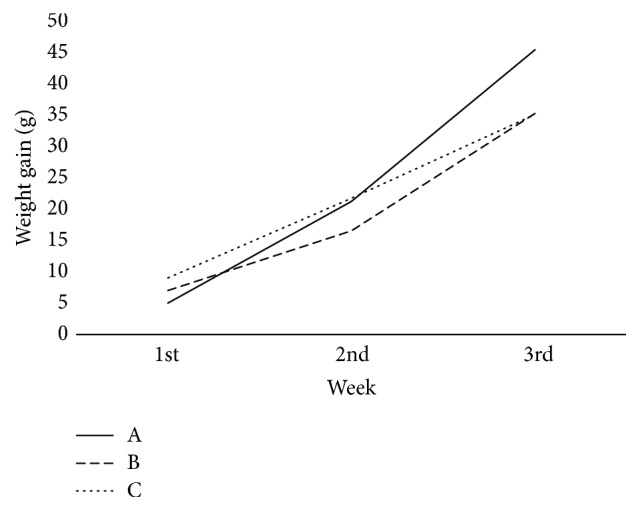
Comparison of rat's body weight gain between three groups. A: high protein only (HP), B: high protein,* Lactobacillus casei* Shirota, and aflatoxin B_1_ (HP + LcS + AFB_1_), C: high protein and aflatoxin B_1_ (HP + AFB_1_).

Rats in group A gained weight throughout the study. Conversely, rats in groups B and C that were gavaged with AFB_1_ had lower weight gain compared to rats in group A (Figure 1). This observation is in agreement with other studies [14, 20]. AFB_1_ can cause weight loss and reduce food consumption by reducing the level of leptin [21], which directly affects the energy balance and body weight gain [22]. In the present study, no significant difference on the body weight gain was observed between group A (58.5 ± 4.6 g) and group C (53.33 ± 7.9 g), as the HP diet enhances detoxification of AFB_1_ [12]. However, rats in groups B (47.67 ± 5.5 g) had significantly lower weight gain (*p* < 0.05) compared to rats in group A. This observation is consistent with a study, where rats supplemented with probiotic* Lactobacillus plantarum* (Lp) had lower weight gain compared to group of rats that consumed high-energy-dense diet only [23]. Besides, it is postulated that rats supplemented with probiotic VSL#3 might have low weight gain due to the production of satiety hormone GLP-1, as the increment of this hormone assists in calories and fat burning [24]. In another animal study, the supplementation of* Lactobacillus paracasei* spp.* paracasei* F19 (F19) had a higher level of Angiopoietin-like 4 (ANGPTL4), which is likely to decrease fat storage [25] and subsequently lead to a reduction in body weight. It is supported by findings from a human intervention study, where a significant decrease in BMI, subcutaneous fat, visceral fat, and waist circumference was observed among subjects in the group supplemented with milk that contained 2 × 10^8^ CFU of probiotics [26]. In addition, supplementation of* Lactobacillus* species caused a significant decrease in body weight and body fat in female subjects [27].

Besides that, probiotic supplementation affects the energy metabolism of the host through the production of short chain fatty acids (SCFA) [28]. A study [29] showed that supplementation of* Lactobacillus salivarius* ssp.* salicinius* JCM 1230 and* Lactobacillus. agilis JCM* 1048 during 24 h in a simulated chicken cecum significantly increased propionate and butyrate formation. In fact,* L. acidophilus* was able to increase SCFAs concentration in SHIME (Simulator of Human Microbial Ecosystem) reactor [30]. An increase of SCFAs is associated with the increment of the circulating concentrations of anorectic gut hormones such as peptide (PYY) and glucagon-like peptide-1 (GLP-1), and these gut hormones have been shown to cause a reduction in energy intake [31]. Other than that, SCFA reduces weight by increasing energy expenditure and enhances fat oxidation and thermogenesis by increasing the rate of oxygen consumption [32].

### 3.2. Liver Function Test

AST and ALT are enzymes of liver function, and elevated activities of these enzymes beyond a certain limit indicate liver lesions or other kinds of damage [33]. Rats in group A had high level of AST and the level was significantly different, compared to rats in group C (Table 1). This result is contradicted with a previous study as AFB1 exposure increased AST level [14]. The dosage of AFB_1_ given to the rats in the present study was similar to that in a study conducted by Nikbakht Nasrabadi et al. [14]. A possible explanation of this finding could be due to the type of diet. As previously mentioned, HP diet enhances detoxification of AFB_1_ [12]. HP diet also has effect on the hepatic enzymes as found in an animal study [34]. Following an HP diet, the liver enzymes enhance the catabolism of amino acid [35]. As the diet has high content of protein, the liver will have to secrete more enzymes for the catabolism of amino acid. Indeed, the increment of AST level between the groups was paralleled to the consumption of food, as rats in group A had higher food consumption than rats in group B and group C.

Despite the higher ALP level in all groups, there were no significant differences between groups (Table 1). Both AST and ALP levels were higher than the normal range (AST: ≤40 IU/L, [36], ALP: 44 to 147 IU/L, [37]) but no liver damage was observed. Additionally, other liver function's biomarkers such as ALT, total protein, and albumin remained in the normal range. The increased level of AST and ALP observed in this study could be the adaptation mechanism of the rats to the HP diet. Adaptation is an intentional response that performs by a body system to a new type of diet consumed, which causes changes in a functional state for better body performance [38]. This was evident in an animal study conducted by Johnson et al. [39], as monkeys that were provided with a high protein diet had elevated ALP level. Hence, changes of liver function's biomarkers, especially AST and ALP, are caused by the consumption of HP diet and are not due to liver damage. However, the effect of HP diet on liver enzymes depends on the percentage of macronutrients used in the experimental diet [40].

### 3.3. Kidney Function Test

The urea levels of the rats in groups A, B, and C were slightly higher than normal range of 5.4 to 7.9 mmol/L, as reported in rats [41] (Table 2). Protein intake causes changes in urea enzyme's cycle activities [35]. Dietary protein will be metabolized to essential and nonessential amino acids [42]. In addition, amino acid will be used to synthesize protein or converted to urea in the liver [42]. The production of urea depends on the amount of dietary protein consumed [42, 43].

In the present study, there was a significant decrease (*p* < 0.05) of urea levels of rats in group B compared to rats in group A. This result agrees with another study [14] that found that the supplementation of LcS reduced the urea level. In contrast, there was no significant difference of urea level between group A and group C, demonstrating that AFB_1_ given to the rats did not affect the kidney function. Slight increase of urea level in this study does not represent kidney disease associated with AFB_1_. In addition, the changes that occurred in renal structure and function are typically a physiological effect and usually happen with increasing dietary protein consumption. A previous study showed that Wistar rats fed with 50% protein showed no abnormalities in renal function or pathology [44]. Besides, the finding by Lacroix et al. [44] showed no adverse effects to sclerotic glomeruli of rat after long-term consumption of diet with 60% of protein. In addition, no association was found between diet and structural changes in the kidney after four years of feeding dogs with 56, 27, or 19% protein [45]. Hence, the changes that occur in renal function are a normal adaptive mechanism in an organism with health kidney [46].

Creatinine is usually used to measure kidney function. Diet plays a vital role that affects creatinine value [47]. In an animal study, HP intake accelerated the progression of renal insufficiency [48]. Another study showed that high daily protein intake will increase the creatinine clearance [49]. With a normal diet, there is a significant increase in creatinine level when the AFB_1_ dose given is 25 ug/kg [16]. However, the present study did not find statistically significant effect on the creatinine level between all groups with the similar dose. In fact, the creatinine value was in the normal range of 30.5 to 114 umol/L [41].

### 3.4. Analysis of AFM_1_ in Urine

AFM_1_ was not detected in urine samples collected from the rats in group A. However, a statistically significant reduction of urinary AFM_1_(*p* > 0.05) was found in group B's rats compared to the rats in group C (Table 3). LcS supplemented to the rats in group B bind AFB_1_ and reduce its absorption in the small intestine [11]. In fact, the reduction of AFM_1_ was about 93% as compared to the rats in group B. In a study by Nikbakht Nasrabadi et al. [14], LcS supplementation caused 85% reduction of an aflatoxin biomarker. The changes in dietary intake can affect the bacteria composition [50]. Diet is the main environmental factor that can influence bacteria diversity and functionality [50–52]. For example, dietary protein affects the overall microbial diversity [53] by increasing beneficial microbiota in the gut [54]. Protein intake provides nitrogen sources for the microbial growth in the colon [55], so there will be sufficient amount of beneficial microorganisms as well as probiotic LcS in group B to adsorb AFB_1_.

Besides, HP intake stimulates the process of hepatic *β*-oxidation [56], which involves CYP2E1 and CYP4A enzymes [54], and a high level of the CYP2E1 enzyme was observed in the hepatocyte-derived cell lines with elevated glutathione (GSH) level. GSH plays a significant role for the detoxification process of AFB_1_ [11] and increased excretion of AFB_1_ from the body. Therefore, HP diet provided to the rats in the present study favored the detoxification of AFB_1_, which was further enhanced by the supplementation of probiotic LcS.

### 3.5. Histopathological Examination

**Figure 2:**
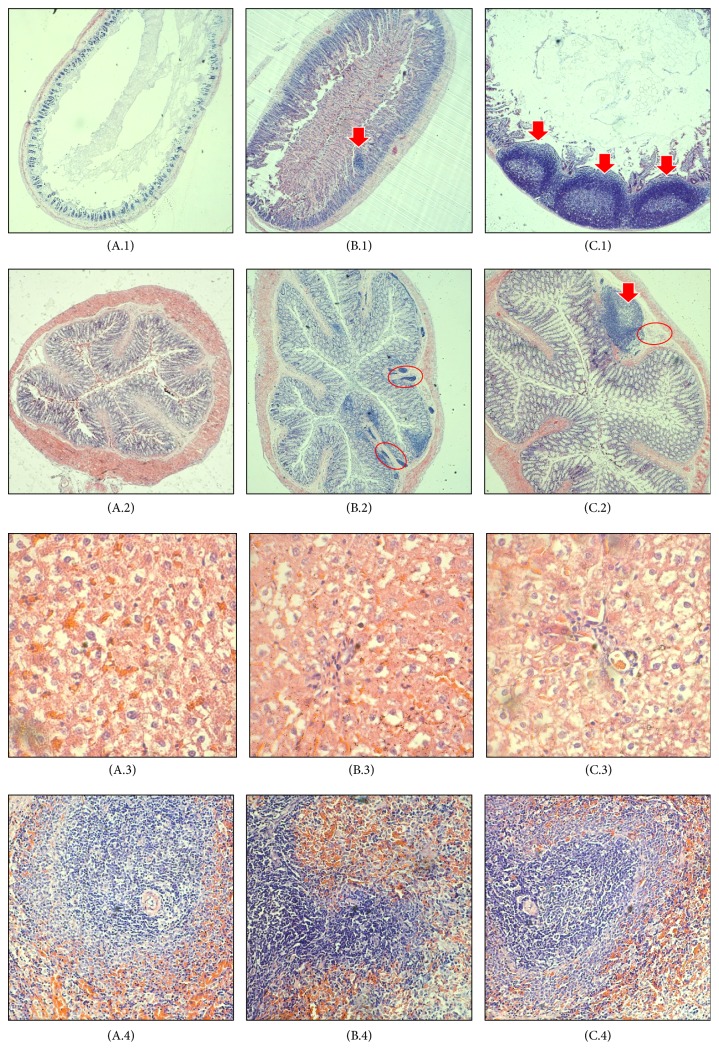
Haematoxylin and eosin staining of small intestine (1), colon (2), liver (3), and spleen (4). A: high protein only (HP), B: high protein,* Lactobacillus casei* Shirota, and aflatoxin B_1_ (HP + LcS + AFB_1_), C: high protein and aflatoxin B_1_ (HP + AFB_1_). In small intestine, tumor-like growth (carcinoma) can be observed in group B and group C. In colon, tumor-like growth (carcinoma) and lymphocytes accumulation (inflammation) can be observed in group C. Group B only showed lymphocytes accumulation (inflammation). However, no prevalent changes have been observed in both liver and spleen. Arrow indicates tumor-like growth; red circle indicates lymphocytes accumulation. *n* = 5.

Histological analysis of H&E stained tissue revealed the histological changes, particularly in small intestine and colon (Figure 2). The small intestine of group A was in a healthy state (A.1) based on the histological observation. After AFB_1_ treatment for 4 weeks, large carcinoma was observed in the small intestine of the AFB_1_-treated group (C.1). Similar carcinoma was observed in group B (B.1), but the carcinoma growth was less in number and smaller in size compared to group C. In the colon, lymphocytes accumulation was observed in both AFB_1_-treated (C.2) and probiotic/AFB_1_-treated (B.2) groups. Lymphocytes accumulation indicates the occurrence of inflammation. However, carcinoma growth is only found in the AFB_1_-treated group (C.2). The results demonstrated the negative effects of AFB_1_ towards small intestine and colon, while such effects can be greatly reversed by the LcS treatment. AFB_1_ is commonly linked to liver cancer. However, in this study, no changes were found in H&E stained liver among the three different groups (A.3, B.3, and C.3). On the other hand, immune dysfunction is also one of the negative impacts from AFB_1_ contamination. Yet, no changes were observed in spleen of all groups (A.4, B.4, and C.4).

As mentioned above, the small intestine is the main site of aflatoxin absorption [57]. Few studies to date have reported the effect of AFB_1_ on intestinal epithelium. Similar to hepatocytes, intestinal epithelial cells express CYPs capable of converting AFB_1_ into the reactive epoxide; therefore AFB_1_ exposure might also promote weight loss through enteropathic effects. It is likely that environmental enteropathy is common in areas where dietary AFB_1_ exposure is endemic [58]. Such enteropathy is associated with histological changes in the small intestine, particularly inflammation and abnormal growth of intestinal cells. Jiang et al. [59] found a decrease in the percentages of T-cells and mRNA expression of cytokines in the intraepithelial lymphocytes (IELs) and lamina propria lymphocytes (LPLs) of the intestine in the AFB_1_ group compared to the control group. In human Caco-2 cells, AFB_1_ affects cell viability and growth, increases lactate dehydrogenase (LDH) activity release, and causes DNA damage [60]. Besides, the number of apoptotic cells in the jejunum and expression levels of Bax and caspase-3 genes were elevated in chickens fed AFB_1_-contaminated diet in comparison with controls [61]. In rodents, AFB_1_ induced intestinal lesions in the duodenum and ileum, which are characterized by a leucocytic and lymphocytic infiltration [62]. The current study demonstrated a significant toxin-induced gut dysfunction syndrome as shown in Figure 2 (C.1). Thus, future studies using this model should monitor gut absorptive and barrier functions of the animals.

Probiotics protect the intestine from xenobiotics, including AFB_1_ [63]. Besides their binding ability towards AFB_1_, probiotics also produce bioactive compounds that provide a significant protection against aflatoxicosis. Bioactive compounds produced by probiotics include antioxidant, anti-inflammatory, anticarcinogenic, and antibacterial compounds [64]. In the case of intestinal cancer prevention, probiotics provide strong antitumor activity via production of SCFA, reduction of colon carcinogenesis enzymes, and reduction of pH [4]. The presence of probiotics also provokes modulation immune system in the gut through alteration of gene expression. Generally, probiotics bind to Toll-like receptors (TLRs) to exert their immunomodulation effects, especially through TLR 2- and 4-dependent manner [65]. AFB_1_ is well known as hepatotoxicant and genotoxicant. However, in this study, no obvious changes were found in the liver of AFB_1_-treated rats (Figure 2: C.3). The immunosuppressive effect of AFB_1_ in this study also could not be observed in H&E stained spleen, as per Figure 2 (C.4). This phenomenon can be explained by the adsorption of AFB_1_ by LcS first occurring in the small intestine, as mentioned earlier. Therefore, intestinal injuries are most prevalent in this study. The intestinal adsorption of AFB_1_ will subsequently reduce systematic exposure [66].

## 4. Conclusion

Overall, this study found that LcS had the ability to bind AFB1 following an HP diet and alleviated the adverse effect of AFB_1_ on body weight and liver and kidney function. In addition, the consumption of LcS in the HP diet also increased the excretion of AFB_1_ metabolite, as AFM_1_ was greatly reduced in the urine. This was confirmed through the reduction of carcinoma occurrence in small intestine and colon for group of rats fed with AFB_1_ and LcS, compared to those fed with AFB_1_ alone. However, this study was limited by the lack of a normal diet group. In addition, broader research is needed to determine the effect of different percentage of protein on the ability of LcS and other probiotics to reduce the negative effect of AFB_1_ as this study only provided the rats with 40% of HP diet. Different macronutrients such as carbohydrates and fat may also have effect on probiotics and aflatoxin metabolism and hence warrant further investigation to determine the efficiency of probiotics as an adsorbent of aflatoxin.

## Figures and Tables

**Table 1:** Biochemistry analysis of rat's blood sample for liver function test.

Parameter	ALT (U/L)	*p* value	AST (U/L)	*p* value	ALP (U/L)	*p* value	ALB (g/L)	*p* value	TP (g/L)	*p* value
Group										
A	48.60 ± 12.13^a^	0.987	196.40 ± 45.23^b^	0.049	164.60 ± 28.43^a^	0.385	31.52 ± 0.89^a^	0.255	69.96 ± 3.70^a^	0.394
B	47.60 ± 10.62^a^		163.80 ± 2.26^ab^		168.40 ± 10.92^a^		30.90 ± 0.65^a^		71.90 ± 1.81^a^	
C	48.00 ± 7.54^a^		142.60 ± 19.62^a^		181.80 ± 15.96^a^		30.32 ± 1.51^a^		69.54 ± 2.56^a^	

A: high protein only (HP), B: high protein, *Lactobacillus casei* Shirota, and aflatoxin B_1_ (HP + LcS + AFB_1_), C: high protein and aflatoxin B_1_ (HP + AFB_1_), ALT: alanine transaminase, AST: aspartate transaminase, ALP: alkaline phosphatase, ALB: albumin, TP: total protein. *p* values were obtained from analysis of variance (ANOVA). Values are expressed as mean ± SD. Values with different superscript letter are significantly different (*p* < 0.05).

**Table 2:** Biochemistry analysis of rat's blood sample for kidney function test.

Parameter	Urea (mmol/l)	*p* value	Creatinine (umol/L)	*p* value
Group				
A	10.82 ± 0.26^b^	0.032	46.2 ± 3.34^a^	0.772
B	10.02 ± 0.73^a^		45.0 ± 2.54^a^	
C	10.10 ± 0.14^ab^		45.0 ± 3.08^a^	

A: high protein only (HP), B: high protein, *Lactobacillus casei* Shirota, and aflatoxin (HP + LcS + AFB_1_), C: high protein and aflatoxin (HP + AFB_1_). *p* values were obtained from analysis of variance (ANOVA). Values are expressed as mean ± SD. Values with different superscript letter are significantly different (*p* < 0.05).

**Table 3:** The concentration of AFM_1_ in urine of aflatoxin-induced rats.

Parameter	AFM_1_ (ng/ml)	*p* value
Group		
B	0.39 ± 0.01	<0.001
C	5.22 ± 0.28	

B: high protein, *Lactobacillus casei* Shirota, and aflatoxin B_1_ (HP + LcS + AFB_1_), C: high protein and aflatoxin B_1_ (HP + AFB_1_). Values are expressed as mean ± SD. *p* value was obtained by independence *t*-test.
